# Remission of Graves’ Disease Through Lifestyle Interventions

**DOI:** 10.7759/cureus.81900

**Published:** 2025-04-08

**Authors:** Pranjali Sharma

**Affiliations:** 1 Endocrinology, Scripps Clinic, La Jolla, USA

**Keywords:** cold water immersion, gluten-free diet, graves' disease, hyperthyroidism, lactose-free diet, lifestyle intervention, physical exercise, stress intervention, selenium

## Abstract

Graves' disease, caused by autoimmune thyrotropin receptor antibody-mediated activation of the thyroid, is characterized by hyperthyroidism, orbitopathy, dermopathy, and acropachy. Graves' disease is treated by anti-thyroid drug therapy, radioactive iodine ablation, or total thyroidectomy. We report the case of a 39-year-old female patient with hyperthyroidism secondary to Graves' disease that was managed through lifestyle interventions only. On presentation, she reported intermittent headaches and had an undetectable thyroid-stimulating hormone (TSH) level. Two weeks later, repeat testing showed an undetectable TSH, free thyroxine (free T4) 2.7 ng/dL (normal range: 0.70-1.48 ng/dL), total triiodothyronine (T3) 5.08 ng/mL (normal range: 0.40-1.93 ng/mL), thyrotropin receptor antibody (TRAb) 20.3 IU/L (reference range: ≤1.75 IU/L), thyroid stimulating immunoglobulin (TSI) 2.3 IU/L (reference range: ≤0.54 IU/L), thyroid peroxidase antibody (TPO) 7.66 IU/mL (reference range: <5.61 IU/mL), confirming hyperthyroidism due to Graves' disease. An iodine-123 (I-123) thyroid uptake and scan showed homogeneously increased iodine uptake (68%) at 4 hours (normal range: 3-16%) and (60%) 24 hours (normal range: 8-25%). The patient was prescribed anti-thyroid drug therapy through methimazole but elected not to take it due to concerns about side effects. She incorporated lifestyle interventions and, over a span of three months, was able to improve clinically and biochemically (TSH: 0.824 mcIU/mL, free T4: 0.77 ng/dL, total T3: 0.73 ng/mL, TRAb: 2.93 IU/L, TSI: 0.26 IU/L, and TPO antibody: undetectable). The lifestyle interventions she pursued included going dairy and gluten-free, ingestion of one to two Brazil nuts daily, regular exercise, mindfulness-based stress management, and cold-water immersion therapy. We review the evidence behind these interventions and discuss the utility of these measures in the management of Graves' disease.

## Introduction

Graves' disease (GD), the most common cause of hyperthyroidism, is an autoimmune disorder of the thyroid caused by thyrotropin receptor antibodies (TRAb) produced by the lymphocytes in Graves' thyroid tissue [1]. It primarily occurs between 20 and 50 years of age and is more common in women than men [1]. GD is characterized by hyperthyroidism, orbitopathy, dermopathy, and acropachy [2]. Symptoms of hyperthyroidism due to GD include fatigue, insomnia, weight loss, palpitations or cardiac arrhythmias, tremors, diarrhea, anxiety, oily skin, hair loss, oligomenorrhea or amenorrhea in females, goiter, hyperreflexia, warm and moist skin. Graves' orbitopathy is characterized by dry eyes, watering, conjunctival injection, periorbital edema, upper lid retraction, proptosis, and restricted extraocular eye movement. Graves' dermopathy, seen in 2-3% of cases, is characterized by marked thickening of the skin, mainly over the tibial area. Graves' acropachy or osteopathy involves subperiosteal bone formation and swelling of the metacarpal bones [2]. Management of GD includes antithyroid drugs (ATDs), radioactive iodine (RAI) ablation, or total thyroidectomy [2]. Lifestyle interventions as a therapeutic option are not commonly used or reported, but may be used in addition to approved first-line therapies. We report a case of a patient with GD who was able to reverse her symptoms and biochemical markers through lifestyle interventions only. She did not pursue current guideline-based therapies due to her concerns about short-term and long-term side effects.

## Case presentation

**Figure 1:**
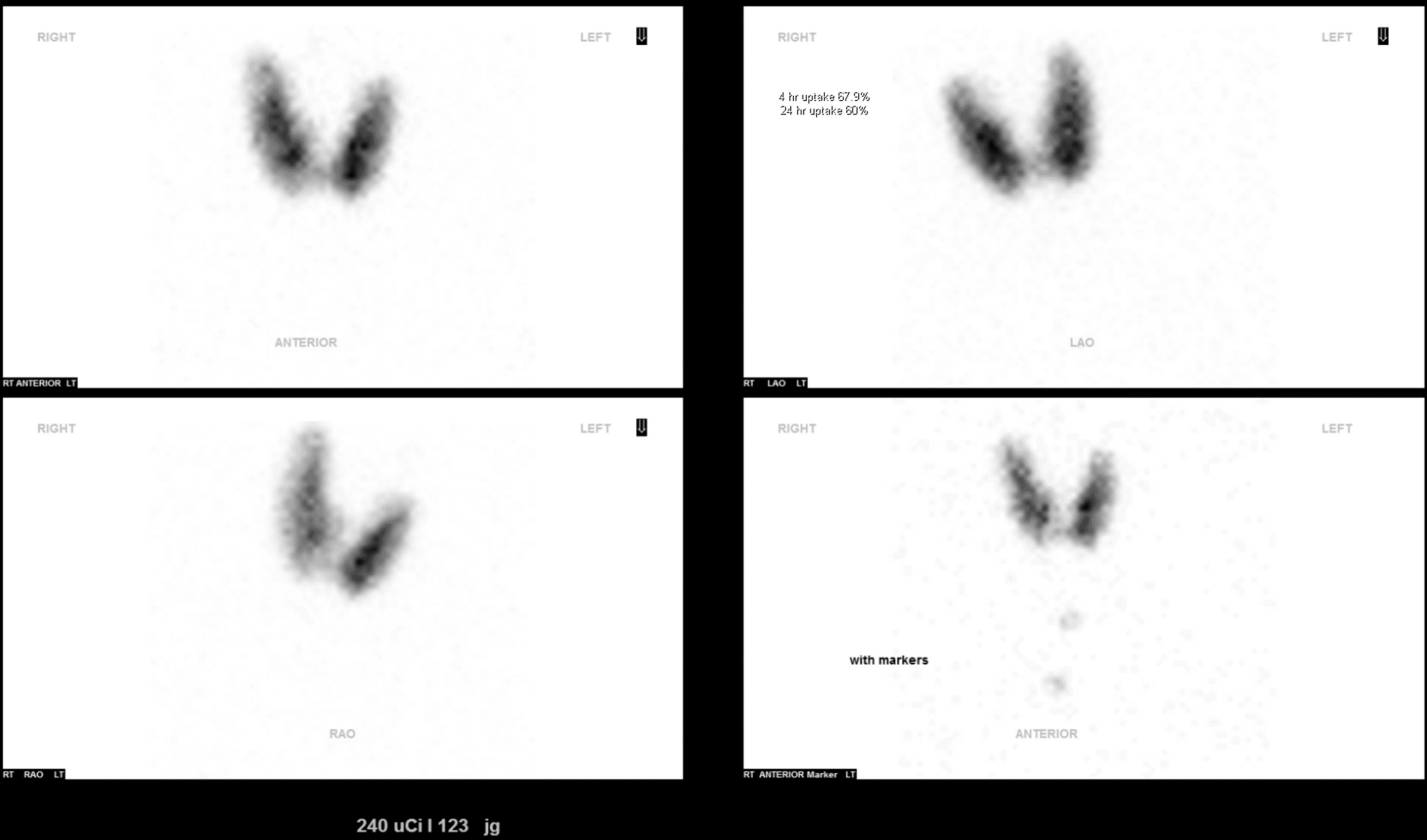
I-123 thyroid uptake and scan.

A 39-year-old female, with no previous medical history or long-term medication use, presented to her primary care physician for intermittent headaches. She reported a normal diet and an overall sedentary lifestyle. Laboratory testing showed an undetectable thyroid-stimulating hormone (TSH) (normal range: 0.350-4.940 mcIU/mL). Repeat testing two weeks later showed an undetectable TSH, free thyroxine (free T4) 2.7 ng/dL (normal range: 0.70-1.48 ng/dL), total triiodothyronine (total T3) 5.08 ng/mL (normal range: 0.40-1.93 ng/mL), thyrotropin receptor antibody (TRAb) 20.3 IU/L (reference range: ≤1.75 IU/L), thyroid-stimulating immunoglobulin (TSI) 2.3 IU/L (reference range: ≤0.54 IU/L), and thyroid peroxidase antibody (TPO) 7.66 IU/mL (reference range <5.61 IU/mL), confirming hyperthyroidism due to Graves' disease (Table 1). An Iodine-123 thyroid uptake and scan showed homogeneously increased iodine uptake (68%) at 4 hours (normal range: 3-16%) and 24 hours (60%) (normal range: 8-25%) (Figure 1).

**Table 1:** Thyroid function test trend. *Abnormal values. TSH: thyroid-stimulating hormone; T4: thyroxine; T3: triiodothyronine; TRAb: thyrotropin receptor antibody; TSI: thyroid-stimulating immunoglobulin; TPO: thyroid peroxidase antibody

Labs	Normal range	Day 1	2 weeks	1.5 months	4.5 months	5.5 months	6.5 months	8 months
TSH	0.35-4.94 mcIU/mL	<0.008*	<0.008*	<0.008*	<0.008*	<0.008*	0.824	2.215
Free T4	0.7-1.48 ng/mL	-	2.7*	3.53*	1.82*	0.86	0.77	0.99
Total T3	0.4-1.93 ng/mL	-	5.08*	4.4*	2.08*	0.85	0.73	0.87
TRAb	≤1.75 IU/L	-	20.3*	-	38.27*	3.73*	2.93*	2.06*
TSI	≤0.54 IU/L	-	2.3*	-	1.06*	0.41	0.26	0.17
TPO	<5.61 IU/mL	-	7.66*	-	4.41*	<3	<3	<3

Patient saw endocrinology one month later and reported unintentional weight loss, heat intolerance, palpitations, and insomnia. Laboratory testing at the time showed undetectable TSH, free T4 3.53 ng/dL, total T3 4.40 ng/mL (Table 1). After discussion regarding various treatment options such as anti-thyroid drug therapy, radioactive iodine ablation, and total thyroidectomy, the patient agreed to take anti-thyroid drug therapy with methimazole. Propylthiouracil was also discussed, but the patient was in favour of trying methimazole. She was prescribed methimazole 30 mg daily and atenolol 25 mg daily. However, after the visit, due to ongoing concerns about side effects, she did not take methimazole. She started atenolol for palpitations and tremors. Within a few weeks, she reported ongoing weight loss but improving heart rate and tremors. Repeat laboratory testing continued to show hyperthyroidism with undetectable TSH and free T4 2.66 ng/dL. The patient was again advised to begin methimazole. We also discussed alternative medications such as cholestyramine, prednisone, and lithium, but the patient was also concerned about the side effects of these drugs. She elected to work on stress management instead, and continued atenolol.

Three months later, labs showed undetectable TSH with free T4 of 1.82 ng/dL, total T3 of 2.08 ng/mL, TRAb of 38.27 IU/L, TSI of 1.06 IU/L, and TPO of 4.41 IU/mL (Table 1). Patient stopped taking atenolol as her palpitations and tremors had resolved. One month later, TSH was undetectable with free T4 of 0.86 ng/dL, total T3 of 0.85 ng/mL, TRAb of 3.73 IU/L, TSI of 0.41 IU/L, and undetectable TPO antibody (Table 1). Repeat testing in the following month showed TSH of 0.824 mcIU/mL, free T4 of 0.77 ng/dL, total T3 of 0.73 ng/mL, TRAb of 2.93 IU/L, TSI of 0.26 IU/L, and TPO antibody undetectable (Table 1). Patient reported improvement in energy and sleep, resolution of palpitations and tremors, no bowel issues, and increased muscle power. On further questioning, she stated that she had been pursuing various lifestyle changes such as a dairy-free and gluten-free diet, ingestion of one to two Brazil nuts daily, daily cardio exercise routine, mindfulness-based stress management, and cold-water immersion therapy once a week. Since the patient was showing clinical and biochemical improvement, she was advised to continue all her lifestyle changes. At eight months, she continues to feel better, and biochemical parameters have shown normal thyroid function with TSH of 2.215 mcIU/mL, free T4 of 0.99 ng/mL, total T3 of 0.87 ng/mL, TRAb of 2.06 IU/L, TSI of 0.17 IU/L, and undetectable TPO antibody (Table 1). She will continue ongoing surveillance with clinical and biochemical evaluation every three months.

## Discussion

This study highlights the useful role of lifestyle interventions in controlling Graves' hyperthyroidism to the extent of eliminating the need for usual forms of treatment. The 2016 American Thyroid Association guidelines indicate that there is no current evidence to support alternative therapies for the management of hyperthyroidism [3]. However, there is an increasing body of research into such alternative therapies. Below, we discuss the evidence behind the lifestyle interventions pursued by our patient.

Dietary modifications

The role of dysbiosis in autoimmune thyroid disorders is being increasingly studied. Disruption of the gut microbiome leads to leaky gut syndrome, characterized by disruption of the intestinal epithelial barrier, increased epithelial permeability, and translocation of bacterial products across the gut [4]. Graves' disease is associated with elevated biomarkers of leaky gut, such as lipopolysaccharides, intestinal fatty acid binding protein, zonulin, D-lactate, and diamine oxidase. Additionally, leaky gut is also associated with impaired thyroid function and poorer quality of life in patients with Graves' disease [5]. Therefore, probiotics and elimination diets have been suggested to restore the gut microbiome and improve the quality of life in GD.

Dairy-free diet

Dairy has been shown to be pro-inflammatory, particularly in those with milk allergy [6]. Patients with untreated GD also have lactose intolerance, which improves after achievement of euthyroidism [7]. Dairy products are also a major source of iodine. Milk iodine concentrations in industrialized countries range from 33 to 534 μg/L, depending on iodine intake of dairy cows, goitrogen intake, milk yield, season, teat dipping with iodine-containing disinfectants, type of farming, and processing [8]. Restricted dairy intake in low iodine diets increases efficacy of RAI for thyroid cancer due to decreased endogenous thyroid hormone production [9]. Therefore, dairy-free diets can improve symptoms of Graves' hyperthyroidism, particularly in those with milk or lactose intolerance.

Gluten-free diet

The association between celiac disease and autoimmune thyroid disorders is partially attributed to shared human leukocyte antigen (HLA) genetic overlap [10]. One study reported that celiac disease is 4.5 times more common in patients with GD than in those without it [11]. Another study showed a slightly higher incidence of GD in patients with celiac disease [12]. Additionally, patients without celiac disease, who do not avoid gluten, tend to have a higher risk of developing autoimmune thyroid disorders [13]. Gliadins in gluten increase the release of zonulin, while wheat germ agglutinin and lectin bind to the glycocalyx of intestinal cells, causing increased intestinal permeability and a pro-inflammatory T-cell and cytokine-mediated response [14]. This immune activation leads to production of antibodies involved in autoimmune thyroid disease, such as GD. Decreased gluten exposure through a gluten-free diet avoids this immune activation, which may be beneficial for GD. Additionally, gluten-free diets have demonstrated decreased PPAR-γ-mediated inflammation [15]. Therefore, gluten-free diets can improve symptoms in GD.

Regular exercise routine

Exercise capacity is compromised in GD due to the effects of hyperthyroidism on the cardiovascular and respiratory systems [16]. Hyperthyroidism-induced myopathy due to increased muscle protein breakdown causes muscle atrophy, more in proximal than distal muscle groups, contributing to decreased muscle endurance [17]. In a study of 22 patients with newly diagnosed GD, those who underwent 16 weeks of resistance training in addition to medical therapy showed an improvement in muscle strength, endurance, and increased muscle mass as compared to those of medical therapy only [17]. A separate study involving 114 patients, the group that underwent a structured exercise program involving walking, strengthening, and stretching, showed an improvement in aerobic capacity, improved fatigue, early attainment of euthyroidism, and medication withdrawal (within six months), and decreased rates of relapse [16]. Exercise activates the sympathetic system and the hypothalamic-pituitary-adrenal axis, which increases circulating cortisol and catecholamine levels. This downregulates the pro-inflammatory TH1 pathway and upregulates the anti-inflammatory TH2 pathway. The TH2 pathway increases humoral immunity, thereby increasing antibody production. It has been suggested that the release of stress hormones increases production of the blocking TRAb, similar to what is seen in pregnancy [16]. Therefore, consistent exercise may favorably alter the course of GD and allow for early remission.

Selenium intake

Hyperthyroidism in GD is a hypermetabolic state that increases oxidative stress, thereby causing thyroid epithelial cell damage, release of autoantigens, and increased TRAb production. Selenium is an essential trace element that forms selenoproteins required for thyroid hormone production and breakdown. These proteins have antioxidant and enzymatic capacity. Their immunomodulatory action occurs through increased cytotoxic T cell and natural killer cell activities. The anti-inflammatory response of selenium is mediated by decreasing tumor necrosis factor alpha and cyclooxygenase-2. Selenium deficiency enhances the oxidative stress in hyperthyroidism. Therefore, selenium supplementation has been studied in the management of GD. In studies of patients with GD in selenium-deficient areas, the addition of selenium to medical therapy achieved euthyroidism more rapidly [18]. The European Group on Graves' Orbitopathy (EUGOGO) randomized control trial also showed that selenium supplementation improved clinical activity scores, decreased eye involvement, and slowed down the progression of thyroid eye disease in mild Graves' orbitopathy [19]. Selenium supplementation should therefore be considered in patients with GD, especially those with Graves' ophthalmopathy. The recommended daily allowance of selenium is 55 mcg in non-pregnant adults, and the maximum daily intake is 400 mcg [4]. Foods such as seafood, meat, poultry, and dairy are good sources of selenium. Brazil nuts contain high amounts of selenium (68-81 mcg/nut). Patients should be advised to avoid ingesting more than one Brazil nut daily due to the risk of heart failure, kidney failure, tremors, and other nervous system problems with increased selenium intake [20].

Stress management

Emotional stress is known to precipitate in GD due to increased levels of glucocorticoids, catecholamines, and pro-inflammatory cytokines, leading to immune system activation [21]. Additionally, T3 plays a role in mental health through its effect on serotonin and noradrenaline [22]. Stress reduction can normalize immune system function, thereby allowing for recovery from GD. Higher remission rates have been seen in GD patients on a longer psychotherapy treatment regimen as compared to a shorter one [22]. A recent case series of 11 patients with GD also describes spontaneous remission after stress relief [21]. Mindfulness-based stress reduction is a popular stress reduction technique and has been shown to decrease mental health issues in GD and other chronic health problems [23].

Cold water immersion

Cold plunge therapy is a newer trend that has been touted to have several health benefits. While there is no specific research evaluating the effect of cold water therapy in GD, it is argued that repeated cold water immersions enhance humoral and cell-mediated immunity through increased catecholamine release, similar to the effect of exercise [24]. Therefore, we hypothesize that cold water immersions may increase the production of blocking TRAb, thereby favorably altering the course of GD.

## Conclusions

It is important to note that ATD, RAI, and thyroidectomy remain the first-line standard therapy for the management of GD. Based on the evidence presented above, certain lifestyle interventions, such as dietary modifications, regular exercise, selenium supplementation, stress management, and cold-water immersion, may help to improve quality of life and remission rates in GD. However, this case report highlights the positive effect of lifestyle interventions alone in one young and healthy patient. It is possible that, while the patient pursued the above lifestyle changes, she was better able to cope with symptoms of hyperthyroidism, as compared to a patient with multiple comorbidities. Additional patient-based research and clinical trials are needed in each of the above lifestyle interventions to be recommended as a standard therapy.
